# Improving Nitrogen and Water Use Efficiency in Intensive Cropping by Optimized Management and Crop Rotations

**DOI:** 10.3390/plants15010007

**Published:** 2025-12-19

**Authors:** Huanxuan Chen, Jiawen Qi, Shangyu Guo, Xinsheng Niu, Robert M. Rees, Chong Zhang, Xiaotang Ju

**Affiliations:** 1 School of Tropical Agriculture and Forestry, Hainan University, Haikou 570228, China; 2 Quzhou Experimental Station, China Agricultural University, Quzhou 057250, China; 3 Crop and Soils, Scotland’s Rural College, Edinburgh EH9 3JG, UK

**Keywords:** manure recycling, N use efficiency, water use efficiency, N surplus, nitrate accumulation, wheat–maize rotation

## Abstract

Nitrogen (N) and water are key resources for crop production and improving the efficiency with which they are used remains a major global challenge in intensive cropping systems. Here, we report how crop yield, N and water use efficiency, N surplus, and economic benefits can be improved from optimized management and crop rotations. A conventional winter wheat–summer maize double cropping (CN/*WM*) rotation in a three-year field experiment in the North China Plain is compared with alternative optimized rotations. The first three optimized treatments were wheat–summer maize rotation with optimized N and irrigation rates, tillage and straw management (ON/*WM*), and partial manure substitution (ONM/*WM*) or biochar addition (ONB/*WM*); the fourth optimized treatment was winter wheat–summer maize–spring maize producing three harvests in two years (ON/*WMM*); and the last was spring maize incorporating green manure during the fallow season for one harvest per year (ON/*GM*). The results showed that the ON/*WM*, ONM/*WM*, and ONB/*WM* had comparable yields to CN/*WM*, but significantly increased N use efficiency by 19–41% and water use efficiency by 13–20% and reduced N surplus to 353–531 kg N ha^−1^ 2yr^−1^. From these three optimized treatments, the ONM/*WM* performed better, with a comprehensive evaluation index of 0.66 and the highest economic benefits. The ON/*WMM* and ON/*GM* treatments also significantly increased N and water use efficiency but resulted in relatively low crop yields and profits; nevertheless, they significantly reduced water use and are suitable for water saving cropping systems. We concluded that optimized management-combined manure with synthetic N fertilization in wheat–summer maize rotations can achieve high crop productivity, environmental, and economic benefits, which contribute to a more sustainable crop production.

## 1. Introduction

Crop production plays a vital role in sustaining human life, with nitrogen (N) and water being essential resources for crop growth and productivity [1]. However, improper N management presents significant challenges, including reduced crop yields and environmental impacts such as nitrate leaching [2,3], greenhouse gas emissions [4,5], and water pollution [6,7]. The misuse or over-use of irrigation water causes water waste and shortage and threatens sustainable crop production, particularly in arid and semi-arid regions [8]. There is therefore an urgent need for novel agronomic and management strategies to improve N and water use efficiency and maintain high crop yields [9,10].

Biochar is an emerging agricultural soil amendment and has been regarded as an effective option to mitigate the threats of climate change and ensure global food security [11,12]. Research has shown that biochar application with reduced N applications significantly increases yield, nitrogen uptake, and NUE [13]. Moreover, biochar’s capacity to improve water retention and nutrient availability has been confirmed in multiple cropping systems, contributing to a more resilient and sustainable crop production [14,15,16]. However, biochar application may alter the soil carbon-to-nitrogen (C:N) ratio, thereby affecting soil fertility and carbon storage [17]. For example, biochar maintained crop yields in a rapeseed-soybean rotation system, but it increased greenhouse gas emissions [18]. It has been suggested that low doses of biochar under low N conditions may lead to an increase in the N_2_O emissions [19]. These findings highlight the need for further research on the long-term effects of biochar under different soil types, weather conditions, and cropping systems, which may optimize the application of biochar and enhance the sustainability of agricultural production.

The combined application of manure and synthetic fertilizers has also gained attention for their ability to balance nutrient release rates, improve soil structure, and reduce nutrient losses [20,21,22,23]. Studies have shown that mixed applications of 50% manure and 50% synthetic fertilizer can significantly increase the yields of maize and wheat [24]. This may be attributed to the combined manure and chemical fertilization that can significantly improve soil fertility by increasing the activity of N-metabolizing enzymes, which then increase crop and total biomass yields [25,26,27]. Partial substitution of fertilizer by manure can improve short-term carbon sequestration rates and reduce net global warming potential, which not only enhances soil carbon levels but also mitigates the GHG emissions [28,29]. Green manures also offer N and other essential nutrients, increasing soil organic matter [30,31], promoting microbial activity [32,33], and improving nutrient cycling [34]. Leguminous green manures add N to the soil by biological N fixation and improve soil water-holding capacity, collectively leading to enhanced NUE and WUE [35,36,37]. Recycling manure and green manure to soil is a particularly important measure to increase crop yield and NUE in regions with low soil fertility [38,39] and needs to be evaluated over a long term rather than in a single year or season, because it needs multiple years to establish a steady state between the crop, soil, environment, and management practices [40].

Cropping system designs further influence NUE and WUE through their impact on nutrient and water dynamics. Many studies have focused on developing new cropping systems to optimize agronomic management, thereby achieving a better match between local resources (land, climate, and soil) and sustainable crop production [9]. They also improve productivity, stability, and N use efficiency by better utilizing available resources and reducing environmental impacts [41,42].

The North China Plain (NCP) is recognized as one of the most intensively cultivated agricultural regions in the world and it produces 23% of China’s cereals [43]. The typical soil is fluvo-aquic (calcareous Cambisol according to WRB classification) and is characterized by low fertility because of its low organic matter concentrations [44]. In this area, a double cropping system of winter wheat followed by summer maize is extensively adopted, resulting in significant increases in grain yields over the past fifty years [45]. However, crop production in the region is associated with low NUE and WUE due to improper nutrient management and overexploitation of groundwater, further causing air and water pollution as well as a sharp decline in the groundwater table [9]. Therefore, improving both NUE and WUE and maintaining a high crop yield is urgently needed in the NCP for sustainable crop production.

In this study, we selected the NCP as the study region. The objectives were (1) to evaluate the yield, N and water use efficiency of the optimized N, and management cropping systems compared with a conventional winter wheat–summer maize double cropping system; (2) to evaluate the effects of biochar, manure, and cropping system designs on the sustainable crop production; and (3) to identify the best management practices in the North China Plain for wheat and maize production.

## 2. Results

### 2.1. Crop Production

**Figure 1:**
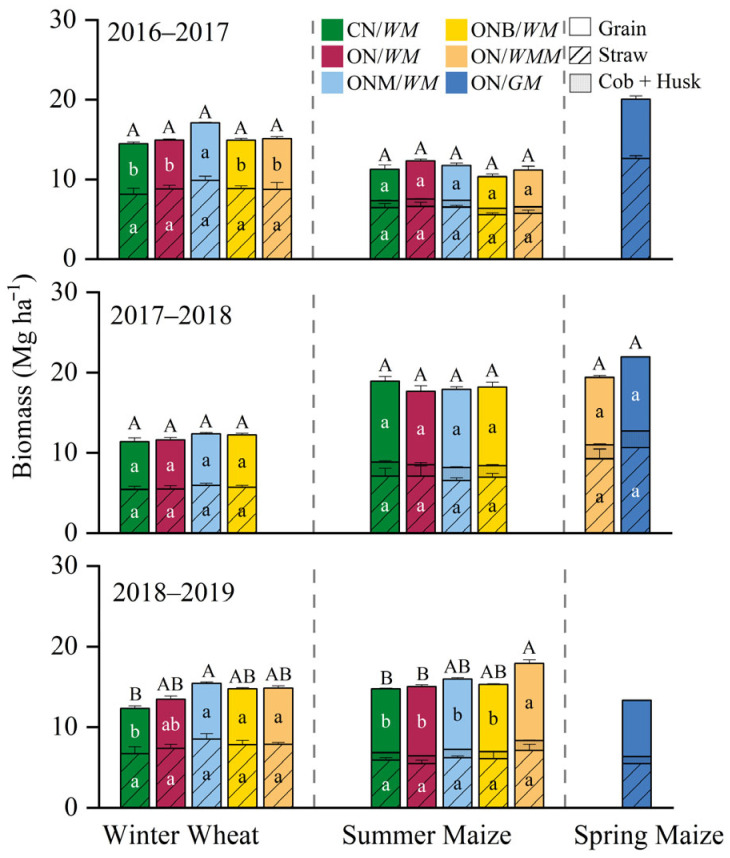
Crop biomasses of different cropping systems in the three-year rotation cycle. CN/*WM*, ON/*WM*, ONM/*WM*, ONB/*WM*, ON/*WMM*, and ON/*GM* represent conventional and optimized N management with winter wheat–summer maize double cropping and two harvests in one year, optimized winter wheat–summer maize double cropping with partial manure substitution and biochar addition and two harvests in one year, optimized winter wheat-summer maize–spring maize with three harvests in two years, optimized spring maize with green manure and one harvest in each year, respectively. The lowercase letters compare the mean biomass of grain, straw, and cob + Husk among cropping systems; upper letters compare the mean aboveground biomass among cropping systems, where different letters indicate significant (*p* < 0.05).

Crop yield varied with years and cropping systems. Winter wheat produced the lowest grain yield of 5.6–6.5 Mg ha^−1^ in CN/*WM*, which was significantly increased by 14% in the ONM/*WM* in 2016–2017 and significantly increased by 23%, 22%, and 23% in ONM/*WM*, ONB/*WM*, and ON/*WMM* in 2017–2018, respectively. No significant differences in grain yield were found in other cropping systems. Summer maize yields averaged 3.9–4.8, 9.1–10.1, and 7.9–9.6 Mg ha^−1^ in the three years, respectively; significant difference was found only in ON/*WMM* when compared with the CN/*WM*, which increased by 21% in 2018–2019. Spring maize had a grain yield of 7.0–9.2 Mg ha^−1^ (Figure 1). The aboveground biomass of winter wheat, summer maize, and spring maize of all cropping systems were in the range of 11.3–17.1, 10.3–18.9, and 14.7–21.6 Mg ha^−1^, respectively; meanwhile, no significant differences were shown between cropping systems with the exception of winter wheat in the ONM/*WM* and the ON/*WMM*, which was significantly increased by 25% and 20% in 2018–2019, respectively (Figure 1). There were no significant differences in the harvest index between different cropping systems in winter wheat, with a range of 0.39–0.47. For maize, the harvest index was significantly lower in ONB/*WM* and ON/*WMM* cropping systems in 2017–2018, but no significant differences were found in 2016–2017 and 2018–2019, with the ranges of 0.34–0.41 and 0.54–0.57, respectively (Figure S2).

### 2.2. N Uptake

**Figure 2:**
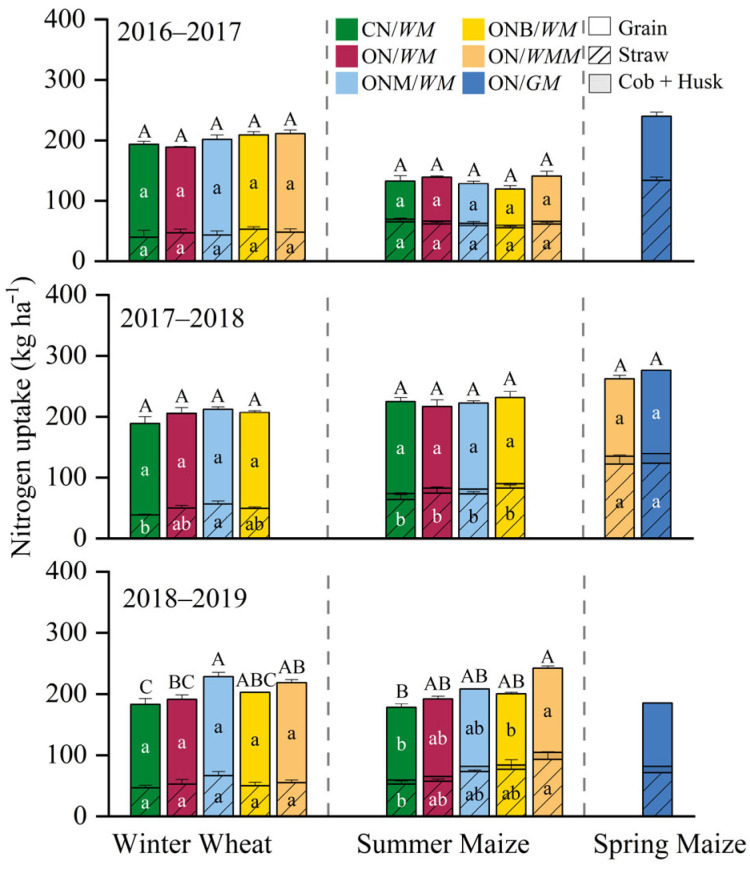
Crop N uptake of different cropping systems in the three-year rotation cycle. CN/*WM*, ON/*WM*, ONM/*WM*, ONB/*WM*, ON/*WMM*, and ON/*GM* represent conventional and optimized N management with winter wheat–summer maize double cropping and two harvests in one year, optimized winter wheat–summer maize double cropping with partial manure substitution and biochar addition and two harvests in one year, optimized winter wheat–summer maize–spring maize with three harvests in two years, optimized spring maize with green manure and one harvest in each year, respectively. The lowercase letters compare the mean N uptake of grain, straw, and cob + Husk among cropping systems; upper letters compare the mean aboveground N uptake among cropping systems, where different letters indicate significant (*p* < 0.05).

In 2016–2017, there were no significant differences in grain N uptake between cropping systems for winter wheat, which was in the range of 136.5–163.2 kg ha^−1^. However, it varied with years in summer maize, which produced a grain N uptake of 60.2–74.9, 134.0–151.0, and 116.6–137.6 kg ha^−1^ in the three years, respectively. When compared with the CN/*WM*, N uptake was significantly larger by 7%, 4%, and 10% in the ON/*WM*, ONM/*WM*, and ON/*WMM* in 2018–2019, respectively. The grain N uptake of spring maize was 103.5–137.1 kg ha^−1^ (Figure 2). The aboveground N uptake of winter wheat was in the range of 183.2–228.5 kg ha^−1^, while in summer maize it varied between 119.4 and 140.9, 216.8–231.8, and 178.4–242.3 kg ha^−1^ in the three years, respectively. N uptake by spring maize was in the range of 185.2–276.4 kg ha^−1^. When compared with the CN/*WM*, for winter wheat it was significantly larger by 5%, 25%, 11%, and 19%, while for summer maize it was significantly larger by 8%, 15%, 12%, and 31% in the ON/*WM*, ONM/*WM*, ONB/*WM*, and ON/*WMM* in 2018–2019, respectively (Figure 2). There were no significant differences in the N harvest index between different cropping systems in winter wheat, with a range of 0.71–0.80. For maize, significant differences were found between different cropping systems, with a range of 0.42–0.67. In 2017–2018, ONB/WM and ON/*WMM* were significantly lower than other cropping systems (Figure S3).

### 2.3. N and Water Use Efficiency

**Figure 3:**
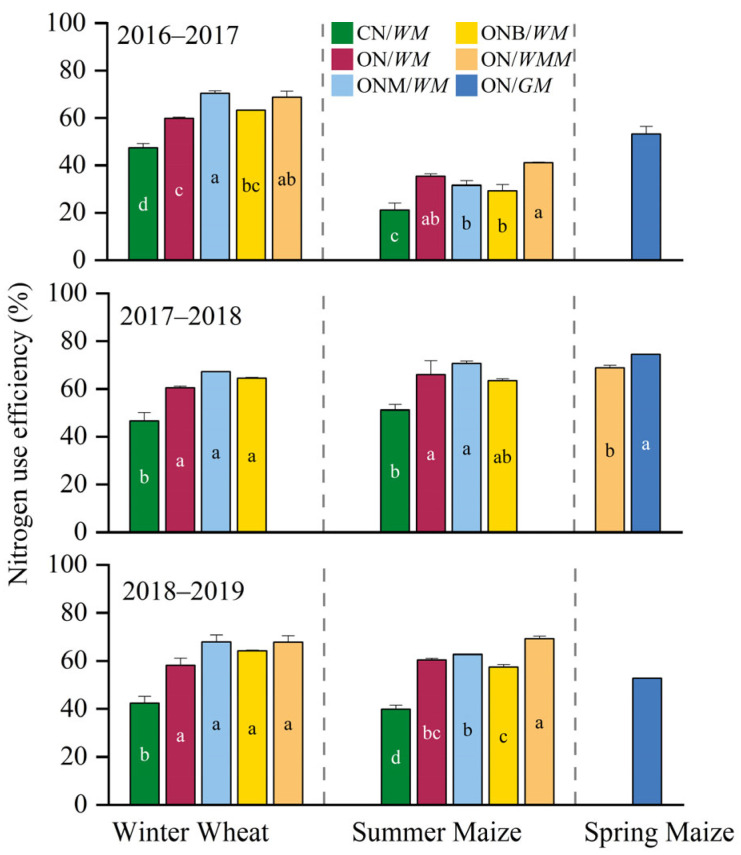
Nitrogen use efficiency of different cropping systems in the three-year rotation cycle. CN/*WM*, ON/*WM*, ONM/*WM*, ONB/*WM*, ON/*WMM*, and ON/*GM* represent conventional and optimized N management with winter wheat–summer maize double cropping and two harvests in one year, optimized winter wheat–summer maize double cropping with partial manure substitution and biochar addition and two harvests in one year, optimized winter wheat–summer maize–spring maize with three harvests in two years, optimized spring maize with green manure and one harvest in each year, respectively. The manure N inputs for the wheat and maize seasons accounted for 40% and 20% of the total manure N input annually, respectively. The lowercase letters compare the N use efficiency among cropping systems, where different letters indicate significant (*p* < 0.05).

The manure N inputs for the wheat and maize seasons accounted for 40% and 20% of the total manure N input annually, respectively. Optimized cropping systems significantly improved N use efficiency (NUE). For winter wheat, NUE in CN/*WM* ranged from 42.4 to 47.6%, which was significantly increased by 26–37%, 44–60%, 33–51%, and 45–60% in the ON/*WM*, ONM/*WM*, ONB/*WM*, and ON/*WMM* cropping systems, respectively. ON/*WMM* achieved a similarly high NUE (67–70%), with an average increase of over 45% compared to CN/*WM*. For summer maize, CN/*WM* showed a low NUE of 21.2–51.1%, while ON/*WMM*, ON/*WM*, and ONM/*WM* increased NUE by 93%, 67%, and 49% in 2016–2017, respectively. NUE was the highest in ONM/*WM* at 70.7% in 2017–2018, representing a 38% increase over CN/*WM*. Optimized cropping systems like ON/*WMM* and ONM/*WM* maintained significantly higher NUE than CN/*WM*, with increases ranging from 43 to 73% in 2018–2019. The NUE of summer maize was significantly increased by 29–61%, 38–57%, 24–44%, and 74–93% in the ON/*GM*, ONM/*WM*, ONB/*WM*, and ON/*WMM* cropping systems compared to CN/*WM* in the three years, respectively. Spring maize showed a NUE of 68.8% and 52.8–74.5% in ON/*WMM* and ON/*GM*, respectively (Figure 3).

There were no significant differences in the physiological efficiency of N use between different cropping systems in winter wheat, with the range of 73.7–82.5, 56.8–62.8, and 67.3–72.9 kg N^−1^ in the three years. For maize, physiological efficiency of N use was significantly lower in the ON/*WMM* cropping systems in 2017–2018, at 69.0 kg N^−1^. No significant differences were found in 2016–2017 and 2018–2019, with a range of 72.1–84.3 kg N^−1^ (Figure S4).

**Figure 4:**
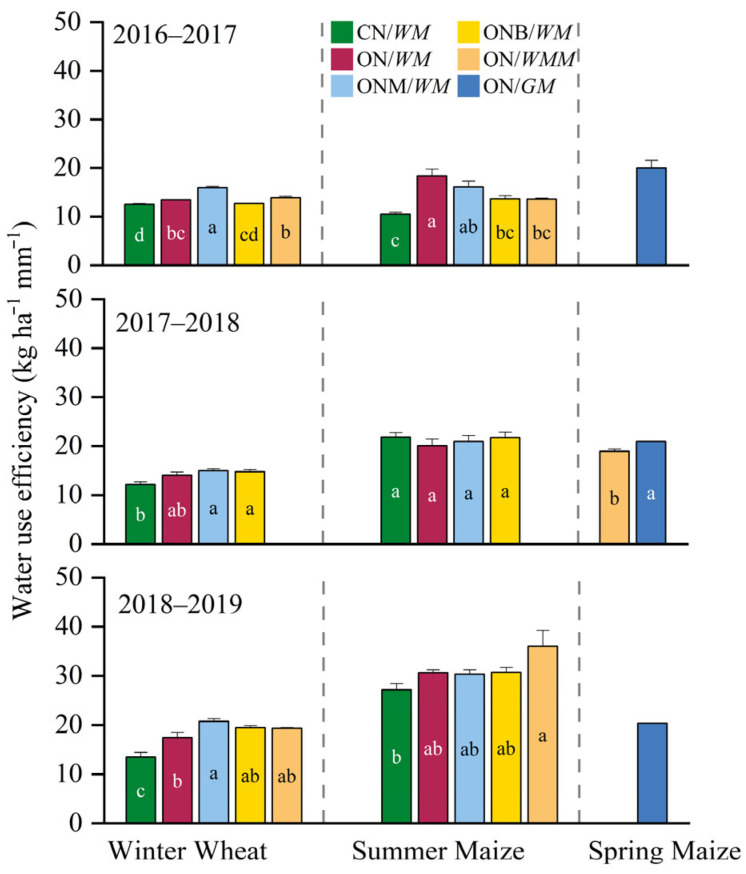
Water use efficiency (kg ha^−1^ mm^−1^) of different cropping systems in the three-year rotation cycle. CN/*WM*, ON/*WM*, ONM/*WM*, ONB/*WM*, ON/*WMM*, and ON/*GM* represent conventional and optimized N management with winter wheat–summer maize double cropping and two harvests in one year, optimized winter wheat–summer maize double cropping with partial manure substitution and biochar addition and two harvests in one year, optimized winter wheat–summer maize–spring maize with three harvests in two years, optimized spring maize with green manure and one harvest in each year, respectively. The lowercase letters compare the water use efficiency between cropping systems, where different letters indicate significant (*p* < 0.05).

Water use efficiency (WUE) varied across years and cropping systems. For winter wheat, WUE in CN/*WM* ranged between 12.2 and 13.5 kg ha^−1^ mm^−1^ across the three years. Compared to CN/*WM*, ONM/*WM* significantly increased WUE by 27% and 53% in 2016–2017 and 2018–2019, respectively. Similarly, ON/*WMM* increased WUE by 11% and 44%, respectively. In 2017–2018, there were no significant differences between the optimized cropping systems, with WUE ranging from 12.2 to 15.0 kg ha^−1^ mm^−1^; however this represented a significant increase of 15–22% compared to CN/WM. For summer maize, WUE in CN/*WM* ranged 10.5–27.2 kg ha^−1^ mm^−1^. In 2016–2017, ON/*WM*, ONM/*WM*, ONB/*WM*, and ON/*WMM* significantly improved WUE by 75%, 53%, 30%, and 29%, respectively, compared to CN/*WM*, but remained stable across all cropping systems, ranging from 20.1 to 21.8 kg ha^−1^ mm^−1^ in 2017–2018. In 2018–2019, WUE was further enhanced in the optimized cropping systems, reaching 30.3–36.0 kg ha^−1^ mm^−1^, which was a 12–32% increase over CN/*WM* (Figure 4).

For winter wheat, CN/*WM* consistently had the lowest IWUE, ranging 20.9–23.7 kg ha^−1^ mm^−1^. Compared with CN/*WM*, ONM/*WM* and ON/*WMM* significantly increased IWUE by 58–71% and 59–66% in 2016–2017 and 2018–2019, respectively. Meanwhile, in 2017–2018, there were no significant differences between cropping systems, with IWUE ranging 23.731.2 kg ha^−1^ mm^−1^. For summer maize, IWUE in CN/*WM* was lower than in optimized cropping systems across all years, at 37.9, 143.7, and 113.1 kg ha^−1^ mm^−1^, respectively. ON/*WMM* had the highest IWUE, which significantly improved IWUE by 53% and 22% compared to CN/*WM* in 2016–2017 and 2018–2019, respectively. Meanwhile, in 2017–2018, IWUE values in all optimized systems were similarly high but there were no significant treatment differences. Spring maize under ON/*GM* showed the highest IWU, ranging 82.7–140.9 kg ha^−1^ mm^−1^ (Figure S5).

### 2.4. Nitrate Accumulation

**Figure 5:**
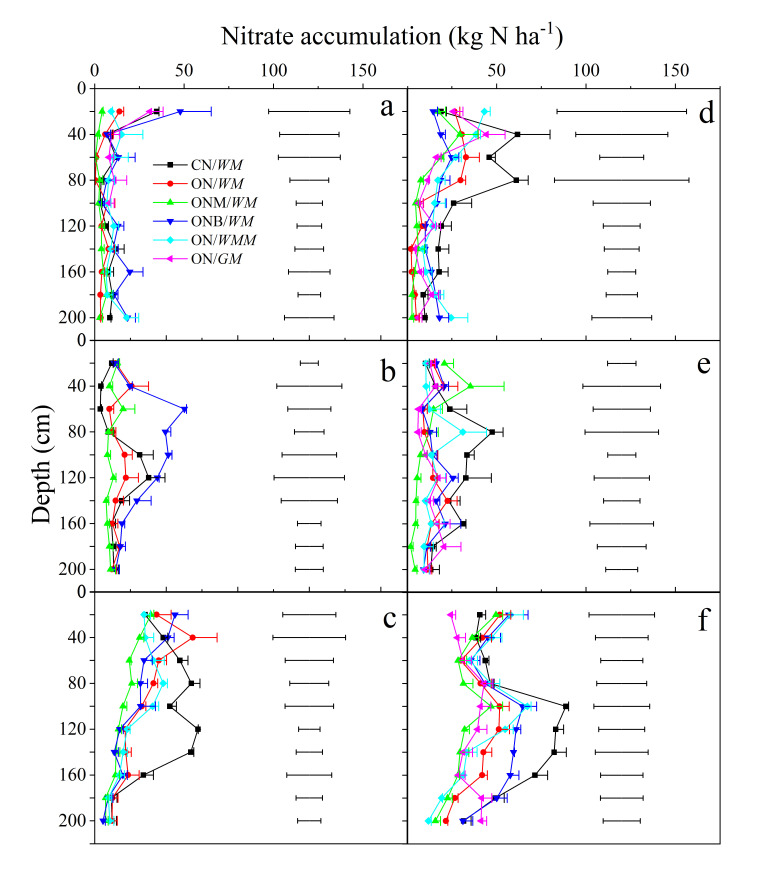
Nitrate accumulation in 0–2 m soil depth after each crop harvest. CN/*WM*, ON/*WM*, ONM/*WM*, ONB/*WM*, ON/*WMM*, and ON/*GM* represent conventional and optimized N management with winter wheat–summer maize double cropping and two harvests in one year, optimized winter wheat–summer maize double cropping with partial manure substitution and biochar addition and two harvests in one year, optimized winter wheat–summer maize–spring maize with three harvests in two years, optimized spring maize with green manure and one harvest in each year, respectively. The horizontal lines in the figure represent the LSD_0.05_ values. (**a**–**c**) represent the nitrate accumulation at wheat harvest in 2016–2017, 2017–2018, and 2018–2019, respectively, and the (**d**–**f**) represent the nitrate accumulation at maize harvest in 2016–2017, 2017–2018, and 2018–2019, respectively.

After harvest, nitrate accumulation gradually migrated to deeper soil layers over the three years (Figure 5). During the wheat seasons, nitrate was mainly concentrated in the 0–20 cm layer in 2016–2017 (4.2–47.8 kg N ha^−1^), then shifted to 40–100 cm in 2017–2018 (3.1–50.2 kg N ha^−1^), and further down to 80–160 cm in 2018–2019 (11.0–57.8 kg N ha^−1^). Significant differences were observed in the 40–80 cm layer in 2017–2018 (ONB/*WM* vs. others) and in the 100–140 cm layer in 2018–2019 (CN/*WM* vs. others). For maize, nitrate accumulated mainly in the 20–80 cm layer in 2016–2017 (7.6–61.7 kg N ha^−1^) and 2017–2018 (6.0–-47.6 kg N ha^−1^), and was significantly different from CN/*WM* and other cropping systems in the 80–100 cm layer in 2017–2018. Meanwhile, in 2018–2019, nitrate accumulation had deepened to 80–140 cm with 29.5–88.8 kg N ha^−1^, showing the following clear system effects: CN/*WM* > ONB/*WM* > ON/*WM* > others, with significant differences between them. After the maize harvest, the ON/*GM* and ONM/*WM* treatment had the lowest nitrate accumulation of 90–98 kg N ha^−1^ in the 0–100 cm soil profile, followed by the ONB/*WM* and ON/*WMM* treatments (136 kg N ha^−1^) and CN/*WM* treatments (154 kg N ha^−1^).

### 2.5. Economic Impacts

Compared to CN/*WM*, the gross outputs in the two-year rotation cycle were increased by 11% and 7% in ONM/*WM* and ONB/*WMM*, respectively, but significantly decreased by 17% and 44% in the ON/*WMM* and ON/*GM*, respectively. The net income was significantly increased by 12% and 23% in the ON/*WM* and ONM/*WM*, respectively, but significantly decreased by 6%, 9%, and 4% in the ON/*WMM*, ONB/*WMM*, and ON/*GM*, respectively. However, the output-input ratio was significantly increased by 18–32% in all optimized cropping systems except ONB/*WMM*, as the differences were caused by the total cost, which mainly comes from the costs of fertilizer and irrigation, while in ONB/*WMM* it was significantly decreased by 18%, which was mainly a consequence of the cost of biochar (Table 1).

### 2.6. Comprehensive Analysis

For the two year-rotation, the CN/*WM* achieved a grain yield of 28.4 Mg ha^−1^ 2yr^−1^, which consumed a large amount of fertilizer and water and resulted in a low N and water use efficiency but a high N surplus. ON/*WM*, ONM/*WM*, and ONB/*WM* had a grain yield and N uptake which was comparable with those CN/*WM* but saved 32–57% of the fertilizer N and 20% of irrigation water; it significantly increased PFPN, N use efficiency, and water use efficiency, with a significantly decreased N surplus. ON/*WMM* and ON/*GM* also saved 50–68% of the fertilizer N and 50–78% of irrigation water, respectively, and were significantly different from ON/*WM* on PFPN, N use efficiency, water use efficiency, and N surplus, but had the lowest grain yield and N uptake of all the cropping systems (Table 2).

**Figure 6:**
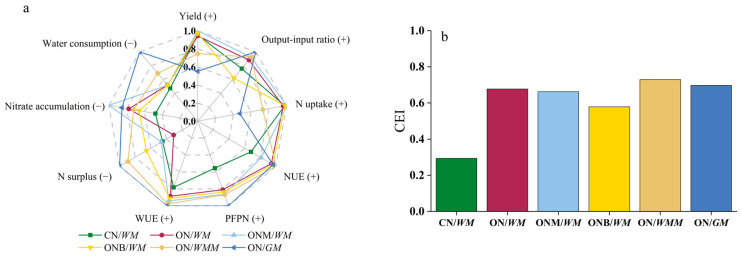
Comprehensive assessments of the different rotation systems. (**a**) Spider plot of multiple objective analysis; (**b**) comprehensive evaluation index (CEI). In (**a**), values are normalized for positive indicators (+), each variable is divided by the maximum of value; for negative indicators (−),−normalization is performed by dividing the minimum value by each variable. CN/*WM*, ON/*WM*, ONM/*WM*, ONB/*WM*, ON/*WMM*, and ON/*GM* represent conventional and optimized N management with winter wheat–summer maize double cropping and two harvests in one year, optimized winter wheat–summer maize double cropping with partial manure substitution and biochar addition and two harvests in one year, optimized winter wheat–summer maize–spring maize with three harvests in two years, optimized spring maize with green manure and one harvest in each year, respectively. NUE: N use efficiency; PFPN: partial factor productivity from applied N; WUE: water use efficiency.

The CEI values were used to assess the synergies of the different crop rotations related to yield, N and water indicators (NUE, PFPN, nitrate accumulation, WUE, N surplus), and output-input ratios. (Figure 6a,b). The results showed that the CN/*WM* rotation had the lowest CEI value of 0.29 (Figure 6b), while the ON/*WMM* rotation had the highest CEI at 0.73, followed by ON/GM rotation of 0.70, ON/WM rotation of 0.68, ONM/*WM* rotation of 0.66, and ONB/WM rotation of 0.58. Significant relationships occurred between the yield, N and water use impact factors across the rotations (Figure S6); water use efficiency was positively corelated with crop yield (r = 0.70), aboveground N uptake (r = 0.36), and N use efficiency (r = 0.32), and PFPN (r = 0.70), but was negatively correlated with N surplus (r = −0.32). N use efficiency was also positively correlated with crop yield (r = 0.68), aboveground N uptake (r = 0.82), and PFPN (r = 0.72), but was negatively correlated with N surplus (r = −0.85).

## 3. Discussion

### 3.1. Crop Productivity

In the winter wheat–summer maize double cropping systems, the optimized ON/*WM*, ONM/*WM*, and ONB/*WM* had crop yields that were comparable with the CN/*WM*, but with a large reduction in fertilizer and manure N inputs of 32% and irrigation by 20% (Table 2); this was because the N rate in the ON/*WM*, ONM/*WM*, and ONB/*WM* treatments were determined by the soil mineral N testing method, which can save fertilizer N by better synchronizing crop N demand and N supply at the critical crop growth stages [46]. Additionally, the mineralization of the added organic residues (straw and manure) in these optimized treatments likely released available N, thereby supporting crop growth. Moreover, optimized treatments used deep plowing and straw return, which can increase crop yield by decreasing bulk density and improving soil structure which in turn promotes the root growth [9,47]. The excess N fertilizer used in the CN/*WM* treatment may have resulted in the loss of yield by increasing vegetative growth [48] and led to lodging and increased susceptibility to diseases or pests [29]. Compared with the conventional cropping systems, the ON/*WMM* and ON/*GM* treatments significantly reduced crop yields by 22% and 42% on the complete rotation cycle basis, respectively (Table 2). This reduction is mainly attributed to fewer harvests in these systems. Unlike the double cropping system, the ON/*WMM* and ON/*GM* treatments included fallow periods, which naturally resulted in lower biomass and grain production and also lead to the substantially reduced N fertilizer and irrigation inputs.

### 3.2. Evaluation of N and Water Use Efficiency

Efficient N use is critical for sustainable crop production [49]. We found that the ONM/*WM*, ONB/*WM*, and ON/*WM* treatments significantly increased N use efficiency (NUE) by 19–41% when compared with CN/*WM*, with no significant differences in NUE among the above three optimized treatments (Table 2). Decreasing N applications is well documented to increase NUE [50,51], although in some N-limited contexts, it may lead to a decrease [52] or remain unchanged [53]. Given that the conventional practice in the North China Plain is mainly characterized by excessive N input, the reduction in N application is widely recognized as a critical strategy to enhance NUE without compromising yield. However, the enhanced NUE observed in our three optimized treatments is not only attributed to the reduced N input; the synergistic interactions of multiple optimized practices also include optimizing N and irrigation rate and timing, straw, and tillage management [54,55,56]. The ON/*WMM* and ON/G*M* cropping system also contributed to a significant increase in NUE by 41–47% (Table 2), which may be attributed to its ability to continuously absorb residual N from deep soil layers during the grain-filling stage [57]. The CN/*WM* treatment involved prolonged reliance on excessive synthetic N fertilizers. Previous studies have widely reported that such practices can lead to soil acidification and decreased microbial activity [58,59], which may collectively contribute to the lower NUE observed in this system.

Compared to CN/*WM*, the ONM/*WM*, ONB/*WM*, and ON/*WM* treatments increased WUE by 13–20% and reduced irrigation by 20% (Figure 4 and Table 2). Among them, the ONM/*WM* treatments were best at improving WUE, although no significant differences were found in some crop seasons (Figure 4). There are several reasons for this—first, long-term combined application of manure and synthetic N fertilizer can improve the physical structure of the soil and increase the water-holding capacity [60]; second, it can increase the soil organic carbon pool and microbial activity, which improves water availability to crops [61,62]; third, the combined application of manure and synthetic N fertilizer can also release N in the later growth stages, which promotes photosynthesis and dry matter accumulation, and thus improves WUE [63]. We found the ON/*WMM* and ON/*GM* treatments further increased the WUE by 25–28%, particularly due to the reduction in wheat production which consumed large amounts of irrigation water.

### 3.3. N Surplus and Nitrate Accumulation

The N surplus in the CN/*WM* was very high (723 kg N ha^−1^) and much higher than the recommended N surplus benchmarks of 320 kg N ha^−1^ (converted from 80 kg N ha^−1^ for per crop seasons) [29,64]. This would likely cause large reactive N losses and negative environmental impacts, including groundwater N contamination and greenhouse gas emissions. It has been demonstrated that reactive N losses increase exponentially with the N surplus increase [65,66]. In contrast, N surpluses from the other optimized treatments were close to the N surplus benchmark of 80 kg N ha^−1^ per crop season except for the ONM/*WM* treatment. Moreover, NUE in the optimized treatments (52–64%) was in the desirable range of 50–90% [64], indicating the management benefits resulting from these treatments.

Soil nitrate accumulation is an important indicator for assessing the performance of N management in agricultural soils [67]. The high nitrate accumulation in the 0–100 cm soil profile will increase the risks of nitrate leaching, thus leading to groundwater N contamination, while the low nitrate accumulation may damage crop growth because upland crops prefer the nitrate N as the N source. The recommended limit for nitrate accumulation in the 0–100 cm is 100 kg N ha^−1^ after crop harvest [68,69]. The CN/*WM* had a high average nitrate accumulation of 155 kg N ha^−1^, while the other optimized treatments reduced it to 90–136 kg N ha^−1^, and thus reduced the risks of N leaching to the deep soil layers and groundwater.

### 3.4. Towards Sustainable Crop Production

We systematically evaluated crop productivity, N and water use efficiency, soil nitrate accumulation and economic benefits across various cropping systems, and C and N management practices in the intensive agricultural region of China. The typical conventional wheat and maize rotation produce grains at the cost of high N surpluses and soil nitrate accumulation with a large consumption of groundwater (Figure 5 and Table 2). In contrast, the optimized C and N management options including ON/*WM*, ONM/*WM*, and ONB/*WM* treatments used the soil N test, with deep plowing, straw return, manure, and biochar application which avoid the above high environmental risks and groundwater consumption. Among them, the combination of manure and synthetic N fertilization (ONM/*WM*) had better performance with the comprehensive evaluation index (CEI) score of 0.66. Although the N surplus in the ONM/WM was relatively high, it contributed to the increase in soil organic N pool [70]. Although the use of biochar (ONB/*WM*) also increased N and water use efficiencies with low N surplus compared to the CN/*WM*, the high cost of biochar led to a low net income and output-input ratio (Table 1 and Table 2), which may hamper its adoption by farmers. The net income in the ON/*WM* treatment was also significantly lower than that in the ONM/*WM*. Therefore, in the wheat and maize rotations, a combination of manure and synthetic N fertilization is a good management practice in the NCP. China has substantial potential in organic nutrients at the amount of over 70 million tons [71]. However, due to limitations in organic fertilizer utilization technologies, the utilization rate remains below 40%, which needs to be improved by transforming agricultural production practices and addressing the technical challenges in the future [72].

We found the alternative cropping systems (ON/*WMM* and ON/G*M*) with three or two harvests in two-year rotation cycles which also achieved high N and water use efficiency with significantly lower crop yield and economic benefits than the CN/*WM*. Despite the reduction in crop yields and profits, it saved groundwater use (particularly for winter wheat with large water consumption), which is important for the NCP and other regions facing the problems of the decline in the groundwater table. Moreover, these alternative cropping systems are also suitable for the regions that need crop fallow, for example, some of the groundwater funnel area in NCP has been selected as a pilot region to implement the “seasonal cropland fallow” to try to halt the decline in the groundwater table [73]. We acknowledge that this study evaluated N efficiency at a system-level rather than quantifying specific N-cycle pathways. Future studies incorporating nitrogen mineralization, N_2_O emissions, and ammonia NH_3_ volatilization would be valuable to further understand the underlying biogeochemical mechanisms by how optimized treatments reduced the N surplus and improved efficiency.

## 4. Materials and Methods

### 4.1. Description of Study Site

A field experiment was conducted from October 2016 to October 2019 at Quzhou Experimental Station (36.87° N, 115.02° E), China Agricultural University, Hebei Province. The experimental area has a temperate monsoon climate, with annual mean temperature of 13.2 °C and mean precipitation of 494 mm. It is characterized by a hot wet summer and cold dry winter (Figure S1). The soil is a calcareous Cambisol (WRB classification) or fluvo-aquic (according to the Chinese soil genetic classification) soil characterized by a high soil pH and low organic carbon content [74]. The initial properties of the 0–20 cm soil layer were measured before the experiment started and the results were as follows: 7.9 of pH (H_2_O), 1.3 g cm^−3^ of bulk density, 13.2 g kg^−1^ of soil organic carbon, 1.0 g kg^−1^ of total nitrogen, 4.8 mg kg^−1^ of NH_4_^+^–N, 20.8 mg kg^−1^ of NO_3_^-^–N, 125.5 mg kg^−1^ of extracted–K, and 43.3 mg kg^−1^ of Olsen–P. All soil properties were measured using the methods described by Bao [75].

### 4.2. Field Experiment Design

A randomized complete block design was designed with six treatments and three replications. The six treatments included different cropping systems and management practices for N, C, irrigation, and tillage as follows: (1) conventional N management with winter wheat–summer maize double cropping and two harvests in one year (CN/*WM*); (2) optimized N management with winter wheat–summer maize double cropping and two harvests in one year (ON/*WM*); (3) optimized winter wheat–summer maize double cropping with partial manure substitution and two harvests in one year (ONM/*WM*); (4) optimized winter wheat–summer maize double cropping with biochar addition and two harvests in one year (ONB/*WM*); (5) optimized winter wheat–summer maize–spring maize with three harvests in two years (ON/*WMM*); and (6) optimized spring maize monocropping, with green manure (rape) planted in the non-maize season, and one harvest in each year (ON/*GM*). The area of each plot was 300 m^2^ (20 m × 15 m).

The N management in the CN/*WM* treatment followed local farmers’ practice in the NCP. In the winter wheat season, 280 kg N ha^−1^ urea-N was applied, with one third applied as the basal fertilizer and two thirds at the shooting stage; in the summer maize season, the same amount of 280 kg N ha^−1^ urea-N was applied, with one third as the basal fertilizer and two thirds at the ten-leaf stage. In the ON/*WM*, ONB/*WM*, ON/*WMM*, and ON/*GM* treatments, the above rates of urea-N were reduced to 200 kg N ha^−1^ for wheat and 180 kg N ha^−1^ for maize (both of the summer and spring maize); the N application time and basal/topdressing ratio were the same as those in the CN/*WM* treatment. N application rates in the ONM/*WM* treatment were the same as those in the ON/*WM* and ONB/*WM* treatments but included a N source from both the manure and urea. A fresh weight of 30 t ha^−1^ manure (0.68–1.87% N) was applied annually in October, and we assumed 40% and 20% of manure-N was mineralized and the gaps between targeted N application rates (200 kg N ha^−1^ for wheat and 180 kg N ha^−1^ for maize) and available manure N were filled by urea. The assumed N mineralization rates were derived from Qiu et al., (2012) [76] in the same region. For the urea N application in the manure treatment, one third of them was applied as basal fertilizer and two thirds during topdressing in wheat season. Half of them was applied as basal fertilizer and the other half at the ten-leaf stage in maize season. A dry weight of 30 t ha^−1^ biochar was applied once in October 2016; we did not assume any significant N release from biochar mineralization to substitute for chemical fertilizer, since its primary role was to improve soil physical properties. The basal fertilizer and the topdressing fertilizer were surface-broadcast before rotary tillage or flood irrigation in the wheat season, while the basal fertilizer was applied in bands near the plant rows and the topdressed fertilizer was applied in bands in the middle of the rows at a depth of 5 cm. The wheat was irrigated with a total of 270 mm according to conventional practice, while for wheat and rape in the optimal practice, the irrigations were performed with a total amount of between 180 and 210 mm. As for spring and summer maize, the irrigations were performed at sowing, between 70 and 90 mm, determined by the weather condition each year (Table S1).

**Figure 7:**
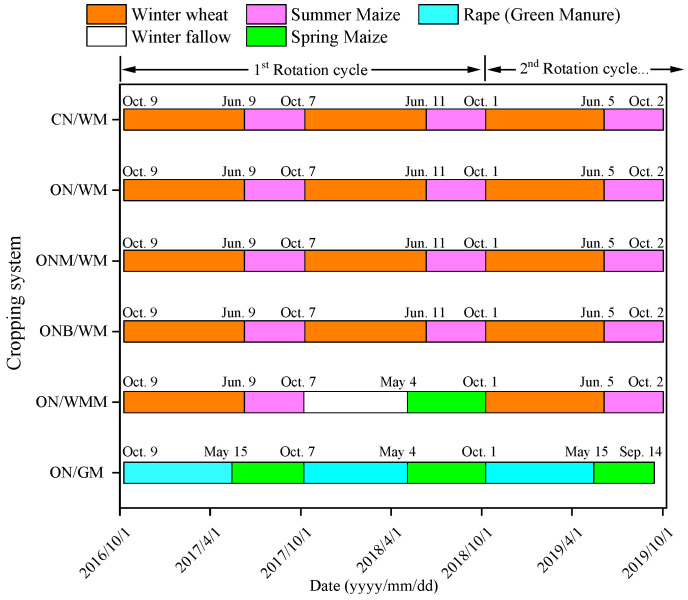
The crop planting sequence during the field experiment. CN/*WM*, ON/*WM*, ONM/*WM*, ONB/*WM*, ON/*WMM*, and ON/*GM* represent conventional and optimized N management with winter wheat–summer maize double cropping and two harvests in one year, optimized winter wheat–summer maize double cropping with partial manure substitution and biochar addition and two harvests in one year, optimized winter wheat–summer maize–spring maize with three harvests in two years, optimized spring maize with green manure and one harvest in each year, respectively.

Crop straw was removed from the field after harvest in the CN/*WM* treatment, while it was shredded by machine and left on the soil surface in other cropping system designs. After harvest, rotary tillage was carried out by a rotary tiller at a depth of 15 cm in CN/*WM* and deep plowing was carried out at a depth of 30 cm in the other treatments, to incorporate the fertilizer, green manure, and straw into the soil. Pests, diseases, and weeds were strictly controlled using local standard practices. In the wheat season, insecticides were sprayed approximately one week after the spring irrigation to prevent early pests, followed by herbicide application in mid-April for weed control. In the maize season, a combination of pre-emergence herbicides and insecticides was sprayed immediately after sowing. Subsequent management included weeding, thinning, and pesticide spraying in early July, followed by supplementary weeding in early August. In addition, to control the pests effectively, granular insecticides were applied at the V12 stage. Further details regarding the implementation and timing of field management operations are shown in Figure 7 and Table S1.

### 4.3. Soil and Plant Sampling and Measurements

Soil samples were collected from the 0–200 cm soil profile at 20 cm intervals at crop maturity for the wheat and maize and sieved through a 2 mm screen. From each sample 12 g was extracted with 100 mL 0.01 mol L^−1^ KCl solution and then used to determine the nitrate concentration by an auto-analyzer (Smartchem 450, KPM Analytics, Villeneuve-la-Garenne, France). Five soil moisture meter sensors (5TE, Campbell scientific, UT, USA) were installed at the soil depths of 10 cm, 30 cm, 50 cm, 70 cm, and 90 cm in each plot to measure the soil water content at a depth of 0–100 cm and the data were collected by a data logger (EM50, Decagon, WA, USA). At maturity, wheat was separated into grain and straw while maize was separated into grain, straw, and cob + husk. The samples were first dried at 105 °C for 30 min and then dried at 70 °C until constant weight. The dry weight of each organ was defined as its biomass; the biomass of the grain at maturity was defined as the grain yield. After that, the samples were weighed to measure the biomass of different organs and sieved through a 0.15 mm screen to measure the N content using the Kjeldahl method [77].

### 4.4. Calculations and Statistical Analysis

#### 4.4.1. Water Use Efficiency

Water consumption was calculated by the variations in the soil moisture content, and water use efficiency was calculated by the grain yield, water consumption, and irrigation rate [78,79].

(1) Water consumption (mm) = 10 × ∑γ_i_ H_i_ (θ_i1_ − θ_i2_) + M + P_0_ + K

where i is the number of soil layer, γ_i_ is the bulk density of the ith soil layer (g cm^−3^), H_i_ is the thickness of the ith soil layer (cm). The soil here denotes the 0–100 cm layer and was divided into five layers, each with a thickness of 20 cm. θ_i1_ and θ_i2_ are the soil moisture contents of the ith soil layer at the start and end of the growth season, respectively. M is the amount of irrigation (mm), P_0_ is the precipitation (mm), and K is the supplemental amount from groundwater of the growth season (mm). The K value is negligible when the groundwater is below 2.5 m from the surface. The groundwater level of the experimental site was below 20 m [80], so K was set to “0” in this study.

(2) Water use efficiency (kg ha^−1^ mm^−1^) = grain yield (Mg ha^−1^)/water consumption (mm) × 1000

(3) Irrigation water use efficiency (kg ha^−1^ mm^−1^) = grain yield (Mg ha^−1^)/irrigation rate (mm) × 1000

#### 4.4.2. N Use Efficiency

The various parameters related to N uptake, harvest index, N use efficiency, soil nitrate accumulation, and N surplus were calculated as follows:

(4) Total N uptake (kg N ha^−1^) = biomass (kg ha^−1^) × crop N content (g N kg^−1^)/1000

(5) Harvest index = grain biomass (kg ha^−1^)/aboveground biomass (kg ha^−1^)

(6) N harvest index = grain N uptake (kg N ha^−1^)/aboveground N uptake (kg N ha^−1^)

(7) Soil nitrate accumulation (kg N ha^−1^) = bulk density (g cm^−3^) × thickness (cm) × soil nitrate concentration (mg N kg^−1^)/10

(8) Partial factor productivity from applied N (kg N^−1^) = grain yield (Mg ha^−1^)/N rate (kg N ha^−1^) × 1000

(9) Physiological efficiency of N use (kg N^−1^) = aboveground biomass (kg ha^−1^)/aboveground N uptake (kg N ha^−1^)

(10) N use efficiency (%) = grain N uptake (kg N ha^−1^)/(fertilizer N (kg N ha^−1^) + biological N fixation (kg N ha^−1^) + N from atmospheric deposition (kg N ha^−1^) + N from seed (kg N ha^−1^) + N from irrigation (kg N ha^−1^)) × 100

(11) N surplus (kg N ha^−1^) = fertilizer N (kg N ha^−1^) + biological N fixation (kg N ha^−1^) + N from atmospheric deposition (kg N ha^−1^) + N from seed (kg N ha^−1^) + N from irrigation (kg N ha^−1^) − Grain N uptake (kg N ha^−1^)

Atmospheric nitrogen deposition data were from Vishwakarma et al. [81], with rates of 17–25 kg N ha^−1^ during the wheat and maize season in the North China Plain (NCP); the estimation of biological N fixation (5 kg N ha^−1^) was from Bouwman et al. [82]. The data of N from irrigation were calculated from the total nitrogen concentration in the irrigation water of 0.64 mg N L^−1^ [83], multiplied by the irrigation volume (mm). The N concentration of wheat, maize, and rape seed were 2.10, 1.60, and 3.48% derived from Gu [84].

#### 4.4.3. Economic Benefit

The economic benefit was calculated by accounting for the input costs of seed, fertilizer, pesticide, labor, and irrigation balanced against the gross output from the products (Equations (12)–(15)). The costs for cropland management and prices of products are shown in Table S2. To evaluate the overall performance of different cropping systems, a comprehensive evaluation index (CEI) was calculated using the Entropy-TOPSIS method based on Yang [43]. The following seven key indicators were selected: yield, output-input ratio, N use efficiency, partial factor productivity from applied N, water use efficiency, nitrate accumulation, and N surplus. Detailed formulas are provided in Supplementary Materials (Text S1, Formulas (S1)–(S12)).

(12) Total cost (¥ ha^−1^) = Input cost of agricultural materials (seed cost + fertilizer cost + pesticide cost) + agricultural operating costs (cost of labor + cost of irrigation)

(13) Gross output (¥ ha^−1^) = grain yield (kg ha^−1^) × product price (¥ kg^−1^)

(14) Net income (¥ ha^−1^) = gross output (¥ ha^−1^) − total cost (¥ ha^−1^)

(15) Output-input ratio = gross output (¥ ha^−1^)/total cost (¥ ha^−1^)

#### 4.4.4. Statistical Analyses

The effects of cropping system (6 levels), crop (3 levels), and year (3 levels), and their interactions on productivity, N uptake, N and water use efficiencies, nitrate accumulation, and surplus were evaluated using a multi-way ANOVA. Duncan’s multiple range test was used to determine differences between the parameters required for ANOVA with a significance level of *p* < 0.05, while Pearson correlations were used to assess the significance of effects in productivity, N uptake, water consumption, harvest index, N and water use efficiencies, nitrate accumulation, and surplus. Statistical analyses were conducted using Microsoft Excel 2016 (Microsoft Corporation, Redmond, WA, USA), SPSS 26.0 software for Windows (SPSS Inc., Chicago, IL, USA), and OriginPro Version 2024b (OriginLab Corporation, Northampton, MA, USA).

## 5. Conclusions

The conventional winter–wheat double cropping system in the North China Plain produces grain with low N and water use efficiency and contributes to high N surpluses and environmental risks. An optimized winter–wheat rotation with or without manure or biochar and included deep plowing, straw return, and soil N test approaches significantly increased N and water use efficiency and reduced N surpluses. Moreover, the combination of manure and synthetic N fertilization had the best performance of the three optimized winter–wheat rotations because of the larger increase in yield and therefore economic benefits. The alternative cropping systems with three or two harvest in two years (and winter wheat–summer maize–spring maize and monocropping spring maize) also significantly increased N and water use efficiency but with a decline of crop yields and profits. Nevertheless, they saved groundwater use and are suitable for regions with low demand for grain production and those that prioritize reducing groundwater use for irrigation.

## Figures and Tables

**Table 1:** Analysis of economic cost and benefit of different cropping systems.

Treatment	Gross Output (10^4^ ¥ ha^−1^)	Input Cost of Agricultural Materials			Agricultural Operating Costs		Total Cost (10^4^ ¥ ha^−1^)	Net Income (10^4^ ¥ ha^−1^)	Output-Input Ratio
		Seed (¥ ha^−1^)	Fertilize (¥ ha^−1^)	Pesticide (¥ ha^−1^)	Labor (¥ ha^−1^)	Irrigation (¥ ha^−1^)			
CN/*WM*	5.4 ± 0.2 c	3585.1	7320.0	974.2	4400.0	2785.0	1.9	3.5 ± 0.0 c	2.8 ± 0.0 d
ON/*WM*	5.6 ± 0.1 bc	3585.1	5700.0	974.2	4400.0	2290.4	1.7	3.9 ± 0.1 b	3.3 ± 0.0 c
ONM/*WM*	5.9 ± 0.1 a	3585.1	6094.6	974.2	4400.0	2290.4	1.7	4.2 ± 0.1 a	3.4 ± 0.0 b
ONB/*WM*	5.8 ± 0.1 ab	3585.1	13,700.0	974.2	4400.0	2290.4	2.5	3.3 ± 0.1 d	2.3 ± 0.1 e
ON/*WMM*	4.5 ± 0.2 d	2484.5	4800.0	974.2	3200.0	1526.2	1.3	3.2 ± 0.0 d	3.4 ± 0.0 b
ON/*GM*	3.0 ± 0.1 e	2016.8	2760.0	488.9	2000.0	827.7	0.8	2.2 ± 0.0 e	3.7 ± 0.0 a

Note: Calculate the economic cost and benefit of different cropping systems from a complete rotation cycle of two years for easy comparison. CN/*WM*, ON/*WM*, ONM/*WM*, ONB/*WM*, ON/*WMM*, and ON/*GM* represent conventional and optimized N management with winter wheat–summer maize double cropping and two harvests in one year, optimized winter wheat–summer maize double cropping with partial manure substitution and biochar addition and two harvests in one year, optimized winter wheat–summer maize–spring maize with three harvests in two years, optimized spring maize with green manure and one harvest in each year, respectively. The fertilizer’s cost of ONB/WM contains the cost of biochar and the fertilizer. The lowercase letters compare the economic cost and benefit between cropping systems, where different letters indicate significant (*p* < 0.05).

**Table 2:** Grain yield, N budget of different cropping systems.

Treatment	N Input (kg N ha^−1^)						Irrigation (mm)	Grain Yield (Mg ha^−1^)	Grain N Uptake (kg ha^−1^)	NUE (%)	PFPN (kg N^−1^)	WUE (kg ha^−1^ mm^−1^)	N Surplus (kg N ha^−1^)
	FN	MN	BNF	DEP	WN	Seed N							
CN/*WM*	1120	0	20	81.5	43.8	10.8	690	28.4 ± 1.0 a	553 ± 16 a	43.3 ± 1.2 d	25.3 ± 0.9 c	15.7 ± 0.1 d	723 ± 16 a
ON/*WM*	760	0	20	81.5	35.2	10.8	555	28.1 ± 0.5 a	544 ± 4 a	59.9 ± 0.5 b	36.9 ± 0.6 b	17.8 ± 0.3 c	364 ± 4 c
ONM/*WM*	476	473	20	81.5	35.2	10.8	555	29.8 ± 0.3 a	565 ± 7 a	51.5 ± 0.6 c	39.3 ± 0.4 b	18.9 ± 0.5 abc	531 ± 7 b
ONB/*WM*	760	0	20	81.5	35.2	10.8	555	29.0 ± 0.5 a	554 ± 7 a	61.1 ± 0.8 ab	38.1 ± 0.7 b	18.3 ± 0.2 bc	353 ± 7 c
ON/*WMM*	560	0	15	81.5	21.9	7.1	345	22.2 ± 0.9 b	436 ± 0 b	63.6 ± 0.0 a	39.6 ± 1.6 b	19.6 ± 0.1 ab	250 ± 0 d
ON/*GM*	360	0	20	81.5	9.5	4.2	150	16.4 ± 0.7 c	291 ± 3 c	61.2 ± 0.7 ab	45.6 ± 2 a	20.1 ± 0.8 a	184 ± 3 e

Note: Calculate the grain yield, N budget of different treatments from a complete rotation cycle of two years for easy comparison. CN/*WM*, ON/*WM*, ONM/*WM*, ONB/*WM*, ON/*WMM*, and ON/*GM* represent conventional and optimized N management with winter wheat–summer maize double cropping and two harvests in one year, optimized winter wheat–summer maize double cropping with partial manure substitution and biochar addition and two harvests in one year, optimized winter wheat–summer maize–spring maize with three harvests in two years, optimized spring maize with green manure and one harvest in each year, respectively. FN: fertilizer N; MN: N from manure; BNF: biological N fixation; DEP: N from atmospheric deposition; WN: N from irrigation; NUE: N use efficiency; PFPN: partial factor productivity from applied N; WUE: water use efficiency. The lowercase letters compare the grain yield and N budget between cropping systems, where different letters indicate significant (*p* < 0.05).

## Data Availability

The data presented in this study are available on request from the corresponding author. The data are not publicly available due to privacy.

## Supplementary Materials

The following supporting information can be downloaded at https://www.mdpi.com/article/10.3390/plants15010007/s1, Text S1: Calculation of comprehensive evaluation index (CEI); Figure S1: Weather conditions from October 2016 to October 2019 during the field experiment; Figure S2: Wheat (a) and maize (b) harvest index of different cropping systems in the three-year rotation cycle; Figure S3: Wheat (a) and maize (b) nitrogen harvest index of different cropping systems in the three-year rotation cycle; Figure S4: Physiological efficiency of N use of different cropping systems in the three-year rotation cycle, (a) wheat, (b) maize; Figure S5: Irrigation water use efficiency (kg ha^−1^ mm^−1^) of different treatments in the three-year rotation cycle; Figure S6: Correlation analysis of yield, nitrogen, and water use impact factors; Table S1: Treatments and field management of experiment; Table S2: Prices and costs for cropland management; Table S3: Soil water consumption (mm) of different treatments in the three-year rotation cycle; Table S4: Multi-way ANOVA analysis for the effects of Cropping System (S), Crop (C), Year (Y) and their interactions of yield, nitrogen, and water use impact factors [85,86,87,88,89,90].
