# Supplementary material for: Improving Nitrogen and Water Use Efficiency in Intensive Cropping by Optimized Management and Crop Rotations
Source: Plants (Basel). 2025 Dec 19;15(1):7. doi: 10.3390/plants15010007 (PMC12787507; doi:10.3390/plants15010007)
Supplement: Supplementary file 1 [file plants-15-00007-s001.zip › plants-3975647-supplementary.pdf]

Supporting materials

Improving Nitrogen and Water Use Efficiency in Intensive Cropping by Optimized Management and Crop Rotations

Huanxuan Chen [1], Jiawen Qi [1], Shangyu Guo [1], Xinsheng Niu [2], Robert M. Rees [3], Chong Zhang [1,*]

and Xiaotang Ju [1,*]

[1] School of Tropical Agriculture and Forestry, Hainan University, Haikou 570228, China

[2] Quzhou Experimental Station, China Agricultural University, Quzhou 057250, China

[3] Crop and Soils, Scotland's Rural College, Edinburgh EH9 3JG, UK

* Correspondence: zhangchong@hainanu.edu.cn (C.Z.); juxt@cau.edu.cn (X.J.)

Text S1: Calculation of Comprehensive Evaluation Index (CEI)

To evaluate the overall performance of different cropping systems, a comprehensive evaluation index (CEI) was calculated using the Entropy-TOPSIS method. The index weight is used to calculate the comprehensive evaluation rank of each crop rotation using TOPSIS:

(1) Normalization of indicators:

$$b_{ij} = \frac{x_{ij} - x_{min}}{x_{max} - x_{min}} \quad \text{(positive indicator)} \tag{S1}$$

$$b_{ij} = \frac{x_{max} - x_{ij}}{x_{max} - x_{min}} \quad \text{(negative indicator)} \tag{S2}$$

where b_{ij} is the normalized value of the jth index in the ith crop rotation (positive indicators include yield, output-input ratio, N use efficiency, partial factor productivity from applied N, water use efficiency; negative indicator include nitrate accumulation and N surplus), x_{ij} is the average value from measured replications of the jth index in the ith crop rotation, and x_{max} and x_{min} are the maximum and the minimum values of a single index, respectively.

(2) Determination of Entropy Weights $(\mathrm{W_j})$: The entropy $(\mathrm{H_j})$ and weight $(\mathrm{W_j})$ for each indicator were calculated as follows:

$$H_j = \frac{-1}{\ln m} \sum_{j=1}^{m} f_{ij} \ln f_{ij} \tag{S3}$$

$$f_{ij} = \frac{b_{ij}}{\sum_{j=1}^{m} b_{ij}} \tag{S4}$$

Where i=1, 2, …, n and j=1, 2, …. m. When $b_{ij} = 0$, $\ln f_{ij}$ is as follows:

$$f_{ij} = (1 + b_{ij}) / \sum_{j=1}^{m} (1 + b_{ij}) \tag{S5}$$

$$W_j = \frac{1 - H_j}{n - \sum_{j=1}^{n} H_j} \tag{S6}$$

(3) Construct a weighted normalized decision matrix (Z): By normalized matrix fij and the weights of each index Wj:

$$Z = \left(W_j \times f_{ij}\right)_{n \times m} = \begin{bmatrix} z_{11} & z_{12} & \cdots & z_{1m} \\ z_{21} & z_{21} & \cdots & z_{2m} \\ \cdots & \cdots & \cdots & \cdots \\ z_{n1} & z_{n1} & \cdots & z_{nm} \end{bmatrix} \tag{S7}$$

(4) Calculation of Euclidean Distances: The positive ideal solution (Z_j^{+}) and negative ideal

solution(Z_j^-) were calculated as:

$$Z_j^+ = \max\,(z_{1j}, z_{12j1}, \dots, z_{nj}) \tag{S8}$$

$$Z_j^- = \min\,(z_{1j}, z_{12j1}, \dots, z_{nj}) \tag{S9}$$

The Euclidean distances (D_i^+ and D_i^-) from the positive ideal solution (Z^+) and negative ideal solution (Z^-) were calculated as:

$$D_i^+ = \sqrt{\sum_{j=1}^{n}\left(Z_j^+ - z_{ij}\right)^2} \tag{S10}$$

$$D_i^- = \sqrt{\sum_{j=1}^{n}\left(Z_j^- - z_{ij}\right)^2} \tag{S11}$$

(5) Calculation of CEI: The CEI for each cropping system was calculated.

$$CEI_i = \frac{D_i^-}{D_i^+ + D_i^-}\,(0 \le CEI_i \le 1) \tag{S12}$$

Table S1 Treatments and field management of experiment

Crops	Managements	CN/*WM*	ON/*WM*	ONM/*WM*	ONB/*WM*	ON/*WMM*	ON/*GM*
2016–2017 winter wheat	Fertilizer application rate ($N:P_2O_5:K_2O$, kg ha^{-1})	280-90-75	200-90-75	200-90-75	200-90-75	200-90-75	-
	Biochar (t/ha)	0	0	0	30	0	0
	Irrigation rate (mm)	270	180	180	180	180	-
	Straw management	Not return	Return	Return	Return	Return	Return
	Tillage after harvest	RT	DP	DP	DP	DP	DP
Maize	Fertilizer application rate ($N:P_2O_5:K_2O$, kg ha^{-1})	280-90-75	180-90-75	180-90-75	180-90-75	180-90-75	180-90-75
	Biochar (t/ha)	0	0	0	0	0	0
	Irrigation rate (mm)	90	90	90	90	90	90
	Straw management	Not return	Return	Return	Return	Return	Return
	Tillage after harvest	RT	DP	DP	DP	DP	DP
2017–2018 winter wheat	Fertilizer application rate ($N:P_2O_5:K_2O$, kg ha^{-1})	280-90-75	200-90-75	200-90-75	200-90-75	-	-
	Biochar (t/ha)	0	0	0	0	0	-
	Irrigation rate (mm)	270	210	210	210	-	-
	Straw management	Not return	Return	Return	Return	-	Return
	Tillage after harvest	RT	DP	DP	DP	-	DP
Maize	Fertilizer application rate ($N:P_2O_5:K_2O$, kg ha^{-1})	280-90-75	180-90-75	180-90-75	180-90-75	180-90-75	180-90-75
	Biochar (t/ha)	0	0	0	0	0	0
	Irrigation rate (mm)	70	70	70	70	70	70
	Straw management	Not return	Return	Return	Return	Return	Return
	Tillage after harvest	RT	DP	DP	DP	DP	DP
2018–2019 winter wheat	Fertilizer application rate ($N:P_2O_5:K_2O$, kg ha^{-1})	280-90-75	200-90-75	200-90-75	200-90-75	200-90-75	-
	Biochar (t/ha)	0	0	0	0	0	-
	Irrigation rate (mm)	270	210	210	210	210	-
	Straw management	Not return	Return	Return	Return	Return	Return
	Tillage after harvest	RT	DP	DP	DP	DP	DP
Maize	Fertilizer application rate ($N:P_2O_5:K_2O$, kg ha^{-1})	280-90-75	180-90-75	180-90-75	180-90-75	180-90-75	180-90-75
	Biochar (t/ha)	0	0	0	0	0	0
	Irrigation rate (mm)	70	70	70	70	70	70
	Straw management	Not return	Return	Return	Return	Return	Return
	Tillage after harvest	RT	DP	DP	DP	DP	DP

Note: CN/*WM*, ON/*WM*, ONM/*WM*, ONB/*WM*, ON/*WMM* and ON/*GM* represent conventional and optimized nitrogen management with a winter wheat-summer maize double cropping system of two harvests in one year, optimized winter wheat-summer maize double cropping system with partly manure substitution and biochar addition and two harvests in one year, optimized winter wheat-summer maize-spring maize with three harvests in two years, optimized spring maize with green

manure and one harvest in each year, respectively. RT denotes rotary tillage and DP denotes deep plowing.

Table S2 Prices and costs for cropland management

Type	Price or cost			Unit	Reference
	2016	2017	2018		
Wheat product	2.332	2.256	2.245	¥ kg^{-1}	[85,86,87]
Maize product	1.643	1.756	1.793	¥ kg^{-1}	[85,86,87]
Wheat seed	1087.35	1100.55	1070.25	¥ ha^{-1}	[85,86,87]
Maize seed	720.75	702.75	685.20	¥ ha^{-1}	[85,86,87]
Synthetic N	4.50	4.50	4.50	¥ kg^{-1}	[88]
Synthetic P	3.00	3.00	3.00	¥ kg^{-1}	[88]
Synthetic K	4.00	4.00	4.00	¥ kg^{-1}	[88]
Manure	432.75	519.15	451.95	¥ ha^{-1}	[85-87]
Biochar	400	400	400	¥ t^{-1}	[89]
Wheat irrigation	1073.70	982.50	875.85	¥ ha^{-1}	[85,86,87]
Maize irrigation	382.80	421.80	429.00	¥ ha^{-1}	[85,86,87]
Wheat pesticide	237.90	237.30	258.15	¥ ha^{-1}	[85,86,87]
Maize pesticide	232.50	245.70	253.80	¥ ha^{-1}	[85,86,87]
Wheat labor cost	1200	1200	1200	¥ ha^{-1}	[90]
Maize labor cost	1000	1000	1000	¥ ha^{-1}	[90]

Table S3 Soil water consumption (mm) of different treatments in the three-year rotation cycle

Treatment	2016–2017			2017–2018			2018–2019		
	Maize	Wheat	Total	Maize	Wheat	Annual	Maize	Wheat	Annual
CN/*WM*	312bc	535a	847a	460a	484a	944a	293b	417a	710a
ON/*WM*	263c	470b	733b	455a	437b	892b	280b	347bc	627b
ONM/*WM*	276c	463b	739b	469a	431b	900b	288b	335c	623b
ONB/*WM*	289c	461b	749b	450a	442b	892b	273b	355bc	627b
ON/*WMM*	385a	458b	844a	443a	-	443c	272b	371b	643b
ON/*GM*	374ab	-	374c	461a	-	461c	342a	-	342c

Note: CN/*WM*, ON/*WM*, ONM/*WM*, ONB/*WM*, ON/*WMM* and ON/*GM* represent conventional and optimized nitrogen management with winter wheat-summer maize double cropping system of two harvests in one year, optimized winter wheat-summer maize double cropping system with partly manure substitution and biochar addition and two harvests in one year, optimized winter wheat-summer maize-spring maize with three harvests in two years, optimized spring maize with green manure and one harvest in each year, respectively. The lowercase letters compare the soil water consumptions between cropping systems, where different letters indicate significant ($p < 0.05$).

Table S4 Multi-way ANOVA analysis for the effects of Cropping System(S), Crop (C), Year (Y) and their interactions of yield, nitrogen and water use impact factors.

ANOVA	GY	BY	NU	WUE	IWUE	NUE	PFPN	NO3-N	Surplus
System	0.178	<0.001	<0.001	<0.001	<0.001	0.431	<0.001	<0.001	<0.001
Crop	<0.001	0.002	0.002	<0.001	<0.001	<0.001	<0.001	<0.001	<0.001
Year	<0.001	0.009	<0.001	<0.001	<0.001	<0.001	<0.001	<0.001	<0.001
S*C	0.466	0.422	0.669	<0.001	<0.001	0.026	0.009	0.169	0.043
S*Y	<0.001	<0.001	<0.001	<0.001	<0.001	0.001	<0.001	0.025	<0.001
Y*C	<0.001	<0.001	<0.001	0.006	<0.001	<0.001	<0.001	0.005	<0.001
S*Y*C	0.790	0.943	0.854	0.002	0.007	0.190	0.142	0.054	0.004

Note: GY: grain yield, BY: biomass yield, NU: aboveground nitrogen uptake, NUE: nitrogen use efficiency, WUE: water use efficiency, IWUE: irrigation water use efficiency, PFPN: Partial factor productivity from applied N, NO_3^--N: nitrate accumulation, surplus: nitrogen surplus.

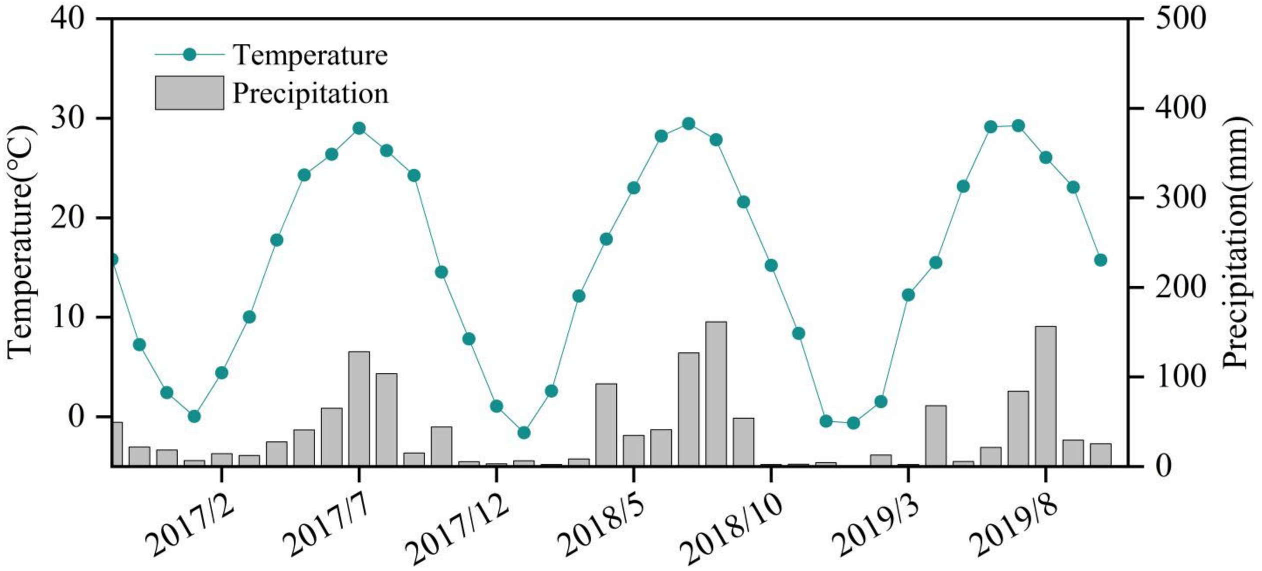

Fig. S1 Weather conditions from October 2016 to October 2019 during the field experiment

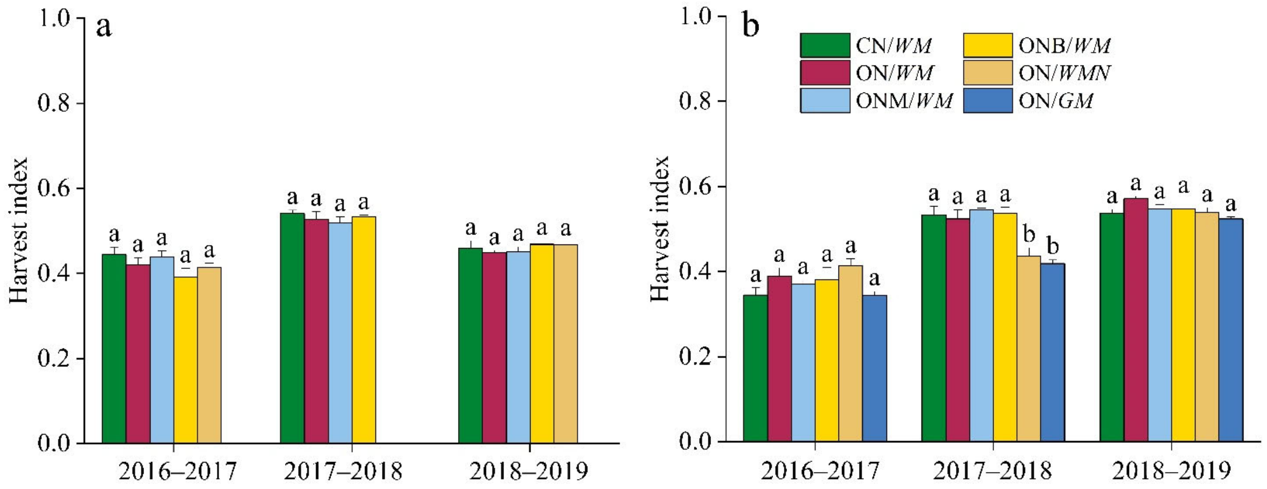

Fig. S2 Wheat (a) and maize (b) harvest index of different cropping systems in the three-year rotation cycle. CN/*WM*, ON/*WM*, ONM/*WM*, ONB/*WM*, ON/*WMM* and ON/*GM* represent conventional and optimized nitrogen management with winter wheat-summer maize double cropping system of two harvests in one year, optimized winter wheat-summer maize double cropping system with partly manure substitution and biochar addition and two harvests in one year, optimized winter wheat-summer maize-spring maize with three harvests in two years, optimized spring maize with green manure and one harvest in each year, respectively. The lowercase letters compare the harvest index between cropping systems, where different letters indicate significant ($p < 0.05$).

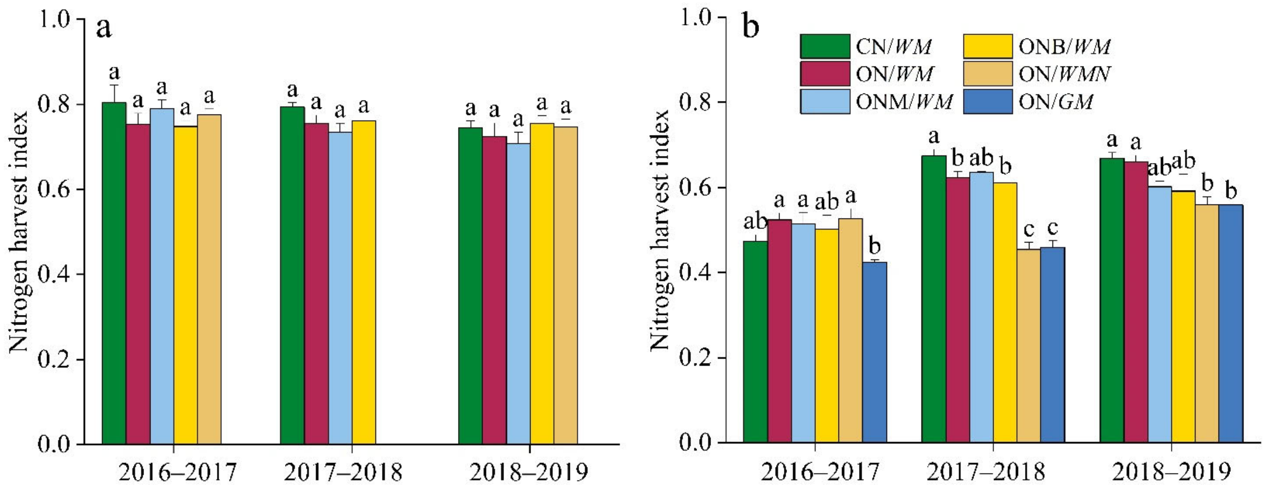

Fig. S3 Wheat (a) and maize (b) nitrogen harvest index of different cropping systems in the three-year rotation cycle. CN/*WM*, ON/*WM*, ONM/*WM*, ONB/*WM*, ON/*WMM* and ON/*GM* represent conventional and optimized nitrogen management with winter wheat-summer maize double cropping system of two harvests in one year, optimized winter wheat-summer maize double cropping system with partly manure substitution and biochar addition and two harvests in one year, optimized winter wheat-summer maize-spring maize with three harvests in two years, optimized spring maize with green manure and one harvest in each year, respectively. The lowercase letters compare the nitrogen harvest index between cropping systems, where different letters indicate significant ($p < 0.05$).

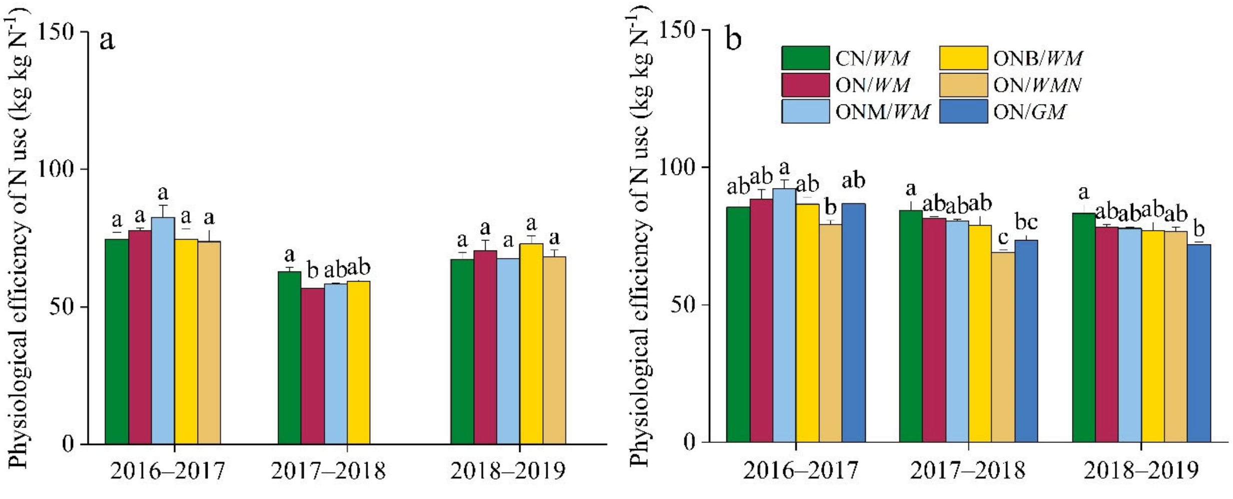

Fig. S4 Physiological efficiency of N use of different cropping systems in the three-year rotation cycle. (a) wheat, (b) maize. CN/*WM*, ON/*WM*, ONM/*WM*, ONB/*WM*, ON/*WMM* and ON/*GM* represent conventional and optimized nitrogen management with winter wheat-summer maize double cropping system of two harvests in one year, optimized winter wheat-summer maize double cropping system with partly manure substitution and biochar addition and two harvests in one year, optimized winter wheat-summer maize-spring maize with three harvests in two years, optimized spring maize with green manure and one harvest in each year, respectively. The lowercase letters compare the physiological efficiency of N use between cropping systems, where different letters indicate significant ($p < 0.05$).

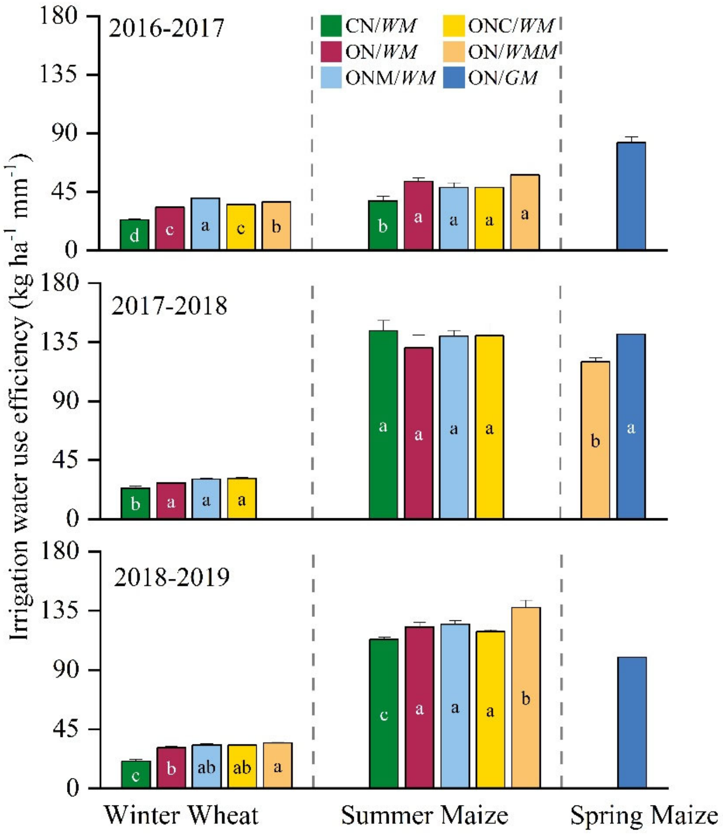

Fig. S5 Irrigation water use efficiency (kg ha $^{-1}$ mm $^{-1}$) of different treatments in the three-year rotation cycle. CN/*WM*, ON/*WM*, ONM/*WM*, ONB/*WM*, ON/*WMM* and ON/*GM* represent conventional and optimized nitrogen management with winter wheat-summer maize double cropping system of two harvests in one year, optimized winter wheat-summer maize double cropping system with partly manure substitution and biochar addition and two harvests in one year, optimized winter wheat-summer maize-spring maize with three harvests in two years, optimized spring maize with green manure and one harvest in each year, respectively. The lowercase letters compare the irrigation water use efficiency between cropping systems, where different letters indicate significant ($p < 0.05$).

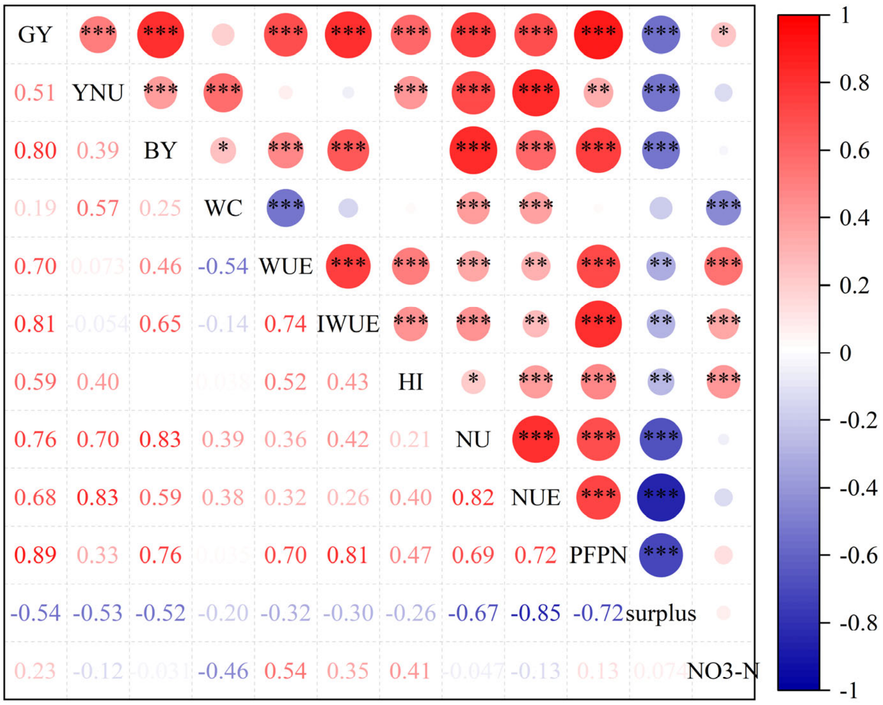

* *P*<=0.05 ** *P*<=0.01 *** *P*<=0.001

Fig. S6 Correlation analysis of yield, nitrogen and water use impact factors. GY: grain yield, GNU: grain nitrogen uptake, BY: biomass yield, WC: water consumption, WUE: water use efficiency, IWUE: irrigation water use efficiency, HI: harvest index, NU: aboveground nitrogen uptake, NUE: nitrogen use efficiency, PFPN: Partial factor productivity from applied N, surplus: nitrogen surplus, NO_3^--N: nitrate accumulation.
